# Species Conservation Profiles compliant with the IUCN Red List of Threatened Species

**DOI:** 10.3897/BDJ.4.e10356

**Published:** 2016-09-01

**Authors:** Pedro Cardoso, Pavel Stoev, Teodor Georgiev, Viktor Senderov, Lyubomir Penev

**Affiliations:** ‡ Finnish Museum of Natural History, University of Helsinki, Helsinki, Finland; § IUCN SSC Spider & Scorpion Specialist Group, Helsinki, Finland; | National Museum of Natural History, Sofia, Bulgaria; ¶ Pensoft Publishers, Sofia, Bulgaria; # Institute for Biodiversity and Ecosystem Research, Sofia, Bulgaria

## The IUCN Red List of Threatened Species

The International Union for Conservation of Nature (IUCN; www.iucn.org) is the world´s largest environmental network, with 1,300 member organizations and relying on the input of about 16,000 experts. It provides knowledge and tools that enable and promote the sustainable development at a global level. Among its many outputs, the Red List of Threatened Species (www.iucnredlist.org) is the most widely known and used, by researchers, politicians and the general public. The IUCN Red List is arguably the most useful worldwide list of species at risk of extinction (Lamoreux et al. 2003). Its usefulness is based on its reliance on a number of objective criteria (IUCN 2012). Threatened species are assessed as either Critically Endangered (CR), Endangered (EN) or Vulnerable (VU), but extinct or non-threatened species are also assessed and listed. Besides extinction risk assessment, the Red List provides a plethora of useful information on each species assessed, including distribution, trends, threats and conservation actions. The quantity and quality of this information allows the Red List to be used in multiple ways, such as to raise awareness about threatened species, guide conservation efforts and funding, set priorities for protection, measure site irreplaceability and vulnerability, influence environmental policies and legislation and evaluate and monitor the state of biodiversity (Gärdenfors et al. 2001, Rodrigues et al. 2006, Baillie et al. 2008, Mace et al. 2008, Martín-López et al. 2009).

Among the almost 2,000,000 named species, 82,845 have been assessed (update 2016-1 of the Red List). This represents less than 5% of species described to date. In particular invertebrates, which constitute the vast majority of species, are still grossly underrepresented (Cardoso et al. 2011a, Cardoso et al. 2011b), but even vascular plants are far from complete (Brummitt et al. 2015). Among the reasons to explain the low representativeness of the IUCN Red List is the lack of experts for many taxa, combined with: 1) the lack of experience of the few available experts on the current system used by IUCN to input data into the Red List (the Species Information System - SIS); and 2) the lack of recognition by academics of red list assessments as peer-reviewed scientific publications, counting towards the "score" of a researcher to its research output in an era of "publish or perish". To help overcoming this knowledge gap, we here propose the use of a specific template in Biodiversity Data Journal as a standard publication venue, both familiar to researchers and recognized by peers, feeding directly into the IUCN Red List of Threatened Species.

## Species Conservation Profiles﻿

Species Conservation Profiles (SCP) are concise treatments of species based on an IUCN-approved template and controlled vocabularies for some of the species characteristics. The Biodiversity Data Journal in collaboration with IUCN SSC members created a workflow that allows for user-friendly authoring, peer-review and publication of SCP via a specially designed template in its authoring platform, the ARPHA Writing Tool (AWT). Apart from the rich editing interface, the tool provides additional functionalities including commenting, replying to comments, importing data from online resources (for example, literature references from CrossRef, PubMed, Mendeley, and occurrence records in Darwin Core format from GBIF, BOLD and iDigBio), versioning, reviewing by external parties during the authoring process, linguistic and copy-editing, building image plates and multimedia, automated technical checking, and others. At the end, the author can submit the profile to the Biodiversity Data Journal just with a click of a button, pass peer-review, and publish it as an open access citable scholarly article within days after acceptance.

The publication will be available in semantically enhanced HTML, PDF and machine-readable XML. Each field in the template is therefore marked as a particular kind of data, being possible to export each species assessment directly to SIS and avoiding duplicate work. Basically, each species assessment published in the journal is fed into the IUCN database and eventually published in the Red List with little extra work. The workflow is expected to play a significant role in experts’ engagement and creates additional incentives for researchers to contribute to the IUCN Red List by publishing new, or updating existing species profiles that can be cited and re-used as any other scholarly article.

It should be noted, however, that the IUCN criteria and guidelines use very specific terminology that in some cases might differ from the standard in conservation science. These include the use of population, sub-population, reduction, continuing decline, extreme fluctuations, severely fragmented, extent of occurrence, area of occupancy and location. To ensure full understanding of concepts, authors are encouraged to refer to the most recent documentation available at the Red List website, namely regarding the categories and criteria (IUCN 2012) and guidelines for their use (IUCN Standards and Petitions Subcommittee 2016). There are also frequent workshops to teach users on the assessments and an official online course (https://www.conservationtraining.org/course/index.php?categoryid=40).

With the availability of this new way of authoring and publishing species conservation profiles compliant with the IUCN Red List of Threatened Species, it is our hope that more experts will feel encouraged to contribute to one of the major goals of IUCN, while at the same time getting the due reward to such important and timely contribution.
